# Approach for characterizing technology- and infrastructure-induced linkages between sustainable development goals

**DOI:** 10.1016/j.xpro.2023.102639

**Published:** 2024-01-13

**Authors:** Magdalena M. Klemun, Sanna Ojanperä, Amy Schweikert

**Affiliations:** 1 Division of Public Policy, The Hong Kong University of Science and Technology, Kowloon, Hong Kong; 2 Energy Institute, The Hong Kong University of Science and Technology, Kowloon, Hong Kong; 3 Oxford Internet Institute, University of Oxford, Oxford, UK; 4 Department of Mechanical Engineering, Colorado School of Mines, Golden, CO, USA

**Keywords:** Energy, Environmental Sciences, Earth Sciences

## Abstract

Technology and infrastructure investments targeting a primary sustainable development goal (SDG) can impact other SDGs. Understanding how linkages are shaped by technology characteristics is vital to design efforts that deliberately leverage co-benefits and mitigate SDG trade-offs. Here, we present a protocol to conceptualize and identify technology-induced linkages. We describe steps for selecting and disaggregating technologies into SDG-relevant impact categories, conceptualizing linkages, and defining scope and scenario. We then detail procedures for computing metrics for a technology’s potential to influence linkages.

For complete details on the use and execution of this protocol, please refer to Klemun et al.^1^

## Highlights

• Technique to evaluate relationships between SDGs
• Steps to apply technique to technology and infrastructure investments
• Screen investment proposals for their potential to connect SDGs

## Abstract (editor highlights)

Publisher’s note: Undertaking any experimental protocol requires adherence to local institutional guidelines for laboratory safety and ethics.

## Graphical abstract

**Figure:**
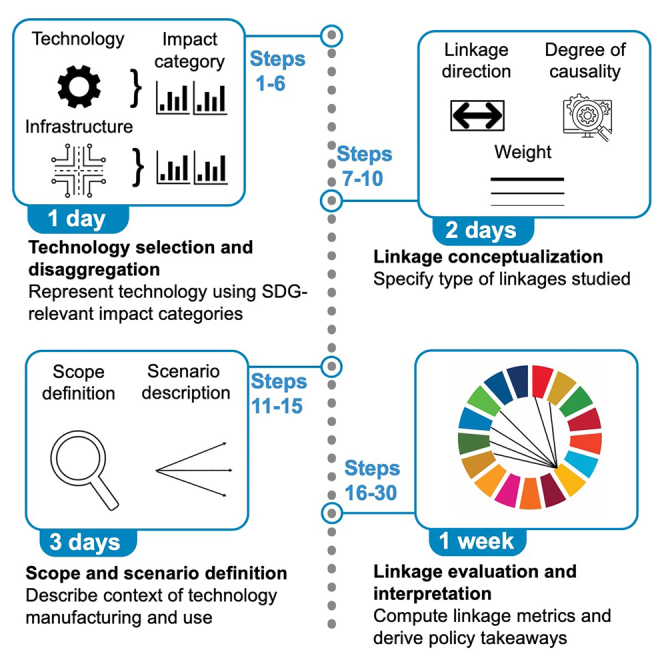

## Before you begin

This protocol can be applied to evaluate the potential of technology and infrastructure investments to induce relationships or “linkages” between SDGs. In the context of this protocol and the underlying study,^1^ “technology” refers to all hardware (machinery or equipment) and soft technology^2^ (processes, services, and associated knowledge and software required for technology design and deployment) that may be used to pursue one or more SDGs. Examples include energy technologies (wind turbines, geothermal power plants), health technologies (blood pressure monitors), water supply technologies (e.g., water purification systems, desalination plants), or general-purpose technologies such as 3D-printing and artificial intelligence applications. Infrastructure refers to physical and non-physical (e.g., organizational) assets, equipment, and other forms of capital serving the provision of energy services (including transportation), as well as education, health, resource management, and other government and social welfare services.^3^ Examples include electricity transmission and distribution lines, roads and refueling stations, rails and train stations, and data centers. Executing the protocol will produce a set of results representing potential linkages between a primary SDG that a technology is used for, and secondary SDGs whose indicators the life-cycle of the technology may also affect.

***Note:*** Application of the protocol is suitable for (but not limited to): scientific studies of technology and infrastructure sustainability impacts, particularly those aimed at expanding beyond standard impact categories (emissions, costs) to relate technologies and infrastructures to the full set of SDGs. The protocol is also suitable for studies of policy co-benefits that aim to expand beyond the most commonly studied co-benefits (e.g., air pollution, health, employment)^4^; evaluations of proposed infrastructure and other development projects in the context of applications for project-specific funding sources (e.g., loans from the International Finance Corporation, the Building Back Better Infrastructure Award by UNECE, direct investment through the International Finance Corporation) or financial support for broader SDG-related policy implementation and crisis management (e.g., International Monetary Fund lending). Several related, context-specific reporting guidelines and tools already exist (e.g., the IFC’s SDG reporting structure,^5^ the UNECE PPP Evaluation Methodology^6^ for proposed public-private partnership projects, etc.) that can be used together with this protocol to better understand technology- and infrastructure related mechanisms shaping SDG linkages); the development of new financing instruments for sustainable development (for example, to identify SDGs affected by financial products developed under the Joint SDG Fund and used for technology funding); assessments of environmental, social, and governance (ESG) performance of organizations (e.g., ESG materiality assessments can draw on the protocol to help identify technology- and infrastructure-related ESG risks); knowledge-sharing and capacity-building initiatives by development banks and other institutions (e.g., the World Bank’s Partnership Fund for Sustainable Development Goals to support strategic initiatives towards SDG17).

## Key resources table

**Table:** 

REAGENT or RESOURCE	SOURCE	IDENTIFIER
**Deposited data**		

Matrices containing technology industry-indicator linkages (Table S1)	DataSpace@HKUST	Database: https://doi.org/10.14711/dataset/FFXY5B

**Other**		

NAICS Code List	NAICS Association	https://www.naics.com/search/
SDG indicator data	UN, SDG Global Database	https://unstats.un.org/sdgs/dataportal
Employment by industry	OECD	https://data.oecd.org/emp/employment-by-activity.htm
R&D expenditure and personnel by sector and enterprise type	OECD Chinese Statistical Yearbook (Tables 20-d7)	https://www.oecd.org/sti/inno/researchanddevelopmentstatisticsrds.htm http://www.stats.gov.cn/sj/ndsj/2020/indexeh.htm
Occupational accidents by industry	International Labor Organization	https://www.ilo.org/moscow/areas-of-work/occupational-safety-and-health/WCMS_249278/lang—en/index.htm
Leadership positions by gender	International Labor Organization	https://www.ilo.org/global/about-the-ilo/how-the-ilo-works/multilateral-system/g20/reports/WCMS_762098/lang—en/index.htm
Life-cycle emissions	GREET, Argonne National Laboratory	https://greet.es.anl.gov/net
	ecoinvent Database	https://ecoinvent.org/the-ecoinvent-database/
Waste statistics	European Union	https://ec.europa.eu/eurostat/web/waste/data/database
	United States Geological Survey	https://www.usgs.gov/centers/national-minerals-information-center/recycling-statistics-and-information
	Chinese Statistical Yearbook (Tables 26-x) Waste Statistics and related World Bank Report	http://www.stats.gov.cn/sj/ndsj/2020/indexeh.htm https://openknowledge.worldbank.org/server/api/core/bitstreams/0f2590e4-0fdb-5f6f-997a-0d736e6cfaaf/content
Air pollution data	World Health Organization	https://www.who.int/data/gho/data/themes/air-pollution
	Urban Air Action Platform	https://www.iqair.com/unep
	United States Environmental Protection Agency	https://www.epa.gov/air-emissions-inventories/2017-national-emissions-inventory-nei-data
National average water use intensity by country	United Nations	https://sdg6data.org/en/indicator/6.4.1

## Step-by-step method details

### Target SDG and technology selection

**Timing: A few days**

This step selects one or more primary SDGs for analysis and a technology or infrastructure type to support progress towards these SDGs.

1. Identify the main SDGs targeted by a particular investment or SDG-related effort.
  ***Note:*** The full list of SDGs and indicators is available at the UN Department of Economic and Social Affairs (https://sdgs.un.org/goals). SDG-specific indicators can be accessed by clicking on each SDG.
  a. Label the target SDGs “primary SDGs”.
    ***Note:*** Primary SDGs can be identified based on funding tools and calls for proposals
  b. Label other SDGs that may be affected by technologies chosen to support the primary SDGs “secondary SDGs”.
    ***Note:*** Secondary SDGs are those affected by co-benefits and trade-offs induced by technologies and infrastructure that support the primary SDGs.
2. Select a technology to support the primary SDG(s).

***Note:***. This can be done by drawing on existing data and literature on technologies’ environmental or socio-economic performance to evaluate whether a technology is suitable to support the primary SDG. Sometimes the technologies eligible for a funding tool may also be specified in a call for proposals.

3. Some applications may involve no primary SDG. In that case, analyze all SDGs, and consider a specific technology as connecting all SDGs for which results (i.e., ‘linkage densities’, see application of impact analysis) are non-zero. In this case, the protocol starts from step 2 and omits step 1.

### Technology disaggregation

**Timing: A few days**

**Figure 1:**
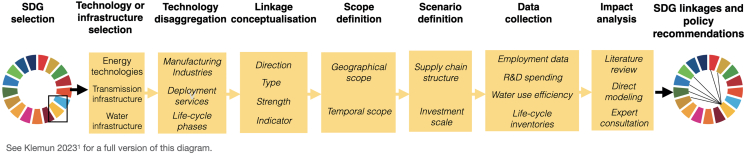
Step-by-step approach to identify and characterize SDG linkages

This step helps select an approach to disaggregate technologies into categories whose effects on SDG indicators can be analyzed (see Figure 1). This step is needed because technologies and infrastructures are higher-order representations of design, manufacturing, deployment, usage, and end-of-life management processes, which cannot be directly related to SDG indicators. These indicators measure concepts related to sub-categories of a technology’s production inputs (e.g., materials usage per unit) or the environmental (life-cycle GHG emissions) and socio-economic impacts of its usage.

4. Identify the secondary SDGs and SDG indicators of interest.

***Note:*** The secondary SDGs are the SDGs on which the impacts of a technology used to support the primary SDG are evaluated. The secondary SDGs could contain all or only a subset of the 16 non-primary SDGs. Depending on the study, the secondary SDGs could be determined by external drivers (e.g., ESG reporting requirements, expectations by the funding agency) or internal drivers such as hypotheses by researchers on potential technology impacts.

5. Select a disaggregation method based on the decisions made in step 4. The goal of step 5 is to match the unit of analysis (whether individual technology industries are used to guide the search for impacts on SDG indicators or other functional units, such as one unit of energy service) to the study’s goal.
  a. If all SDGs are labeled secondary SDGs, choose an approach that can be consistently applied across technologies and SDGs. Disaggregating technologies into industries and services used over their life-cycle^1^ is one option.
  b. If only a subset of SDGs is of interest, consider simplifying the technology disaggregation step by analyzing only technology impact categories directly relevant to the secondary SDG indicators.
    i. For example, if the secondary SDGs are environment-related SDGs such as SDG13, 14, and 15, collect emissions and other life-cycle data rather than disaggregating technologies into industries and services. In that case, the unit of analysis is a unit of service provided by the technology, rather than additional investments in industries and services.
6. Apply the chosen disaggregation method. This step produces a list of technology sub-categories for which impacts on SDG indicators can be analyzed.
  ***Note:*** The technology sub-categories (e.g., industries and services used to manufacture and deploy a technology, see Figure 3 in Klemun 2023^1^ and Table S1 (Technology SDG network), tab 'Matrix for Figure 4/5′, Columns A and B referenced in key resources table under Deposited Data) will later be used to generate keywords for the search for documented, SDG-indicator-relevant impacts in the literature.
  a. If using the approach in Klemun 2023,^1^ search for peer-reviewed bills of materials^7^^,^^8^^,^^9^ enlisting the components and materials that constitute a typical unit of the selected technology. Then, based on a list of components, review the relevant lists of industries in the North American Industry Classification System^10^ (NAICS, or other industry classification systems^11^^,^^12^) for each part of the technology life cycle (design, manufacturing, deployment, operations, end-of-life management) to assess their relevance.
  b. If using life cycle analysis (LCA), consider all impact categories separately and map them onto individual SDGs by comparing SDG indicators to impact categories.
    i. For example, the LCA impact categories climate change (SDG13), land use (SDG15), and resource consumption (SDG12) can be used directly to analyze impacts on SDG indicators. Example of LCA tools include openLCA (https://www.openlca.org), which is a free, open-source software, and commercial tools such as SimaPro (https://simapro.com). An overview of commercial and free LCA databases can be found at https://nexus.openlca.org/databases. For high-level analyses not done in a particular country of technology usage context, protocol users may want to use LCA results from harmonization or meta-studies, as done in Klemun 2023. For energy technologies specifically, comprehensive harmonization studies have been done by the U.S. National Renewable Energy Laboratory^13^ (wind, solar PV, concentrating solar, nuclear fission, natural gas, coal) and by several university-based research teams.^14^^,^^15^
    ii. Other LCA impact categories can be mapped on an SDG based on thematical classifications but do not directly affect SDG indicators (e.g., ‘ozone depletion’ and ‘human toxicity’ to SDG3). Unmatched impact categories can expand the set of indicators considered under specific SDGs.
      **CRITICAL:** Choose a disaggregation method that is a suitable fit for the SDGs and SDG indicators considered in your study. Broad-scope studies of all SDGs often require disaggregation of technologies into industries; narrower studies can use simpler approaches.

### Linkage conceptualization

**Timing: A few days**

This step helps select a set of linkage attributes (direction, type of association between investments and indicators, linkage weight) to define the study’s unit of analysis.

7. Determine the linkage directions considered in the study (either uni-directional or bi-directional).

***Note:*** Uni-directional means that the impacts of technologies on SDG indicators are studied, but not feedbacks of changes in SDG indicators on technologies (i.e., capturing only first-order effects of technology investments). They do not capture, for example, higher demand for energy services due to adoption of lower emission technologies (rebound effect).

Bi-directional means that both technologies’ impact on indicators, and indicator impacts on technologies are considered. For example, by raising income levels, the employment impacts of technology investments can shift consumption patterns and increase demand for larger-scale technologies (e.g., larger vehicles). Considering bi-directional linkages better matches the reality that technology investments do not occur in isolation but typically have a broad set of impacts that can lead to changes in a technology or investment itself.

8. Decide whether SDG linkages are conceptualized as functional, correlational, or both.

***Note:*** Functional linkages arise from industries and services strictly required to deliver a technology. Calling a linkage ‘functional’ means that some effect on an SDG indicator is almost an inevitable consequence of investing in the technology because the life cycle of the technology could not be completed without the mining and processing of materials, the manufacturing, delivery, and installation of components, the use of the final product, and end of life management. In contrast, secondary effects (e.g., effects of additional employment on consumption behavior) or other effects may correlate (see next paragraph) with technology investments but are not preconditions for it.

Correlational linkages indicate an observed association between technology impacts and SDG indicators, but not necessarily a causal relationship. Correlational linkages can provide a window into a broader set of possible technology effects on SDGs, even if these effects cannot (yet) be explained. Considering correlational linkages can thus open the door for future studies. Conversely, it can also introduce linkages driven by other factors rather than technology and infrastructure investments. Additionally, the need for data collection and an open-ended literature search on possible associations between technology investments and SDG indicators will be higher, as more than engineering knowledge and market expertise will be required to identify all possible associations.

9. Decide whether SDG linkages are weighted or unweighted.
  ***Note:*** Weighted linkages will require a methodology to evaluate the strength of individual linkages (e.g., how strongly a technology affects an SDG indicator). Direct modeling of indicator changes is one approach for linkages where historical data are available for model calibration and change mechanisms are understood and can be modeled (e.g., emissions reductions from the displacement of high- with low-carbon electricity); inviting experts to rate the linkages derived from the literature or consulting stakeholders in focus groups are other approaches.
  Unweighted linkages can be determined by defining one or more criteria for when a linkage is positive, negative, or considered negligible. For example, positive linkages indicate that technology investment will positively influence a particular SDG indicator (e.g., reduce GHG emissions or increase employment). In contrast, negative linkages indicate the opposite effect.
  a. If studying weighted linkages, choose a normalization method.
    ***Note:*** Technologies do not affect SDG indicators “starting from scratch” but rather starting from the current indicator levels or a future level of estimated indicator level (in prospective studies). Normalizing indicator changes (e.g., to historical trends) and considering context-specific priorities is thus essential to meaningfully compare linkages. For instance, a small percent change in some indicators (e.g., GDP growth and other growth rate indicators; R&D expenditure as a proportion of GDP; indicators expressed in number of countries) may be broadly considered more influential than the same change in other indicators; stakeholder values are also important to consider.
10. Select the metrics that will be used to evaluate SDG linkages.

***Optional:*** Consider additional linkage attributes relevant to particular research communities, private and institutional actors investing in technologies, or non-governmental organizations monitoring technology investment. For example, distinguishing scale-invariant from scale-variant linkages may be relevant for a better understanding of how much investment is needed to generate specific SDG-related impacts. Any investment in energy technologies will affect land use metrics (SDG15), for example, since one cannot construct a power plant without using space. In contrast, small-scale investments in a certain technology may not trigger R&D investments by companies (SDG9) as smaller capacity targets may be met using current technology. Similarly, one may want to distinguish varying from more permanent linkages to separate temporary SDG effects from long-term impacts.

**CRITICAL:** Choose a linkage conceptualization that matches your research questions. For example, if the goal is to focus on only a subset of SDGs and choose between technologies providing a similar service, weighted linkages may be more meaningful than unweighted linkages. Different technologies can have similar unweighted linkage densities for the same SDG, as shown in Klemun 2023^1^ (with larger differences across SDGs than across technologies suitable for the same SDG).

### Scope definition

**Timing: A few days**

This step helps define the geographical and temporal scope for analyzing linkages, e.g., whether linkages are considered in a particular region and during a specific period. Accurately defining the scope of the analysis is important because two SDGs may be coupled only in a particular region^16^ (where, e.g., air pollution or employment impacts occur), or the degree of coupling may fluctuate over time, depending on a country’s development stage.^17^

11. Define the geographical scope of the study.
  a. Specify where the impacts of technology investments on SDG indicators will be studied.

***Note:*** The narrower the geographical scope (linkages in one organization, one city, one country), the more the study may need to rely on proxy data (e.g., because no empirical data on past employment impacts of a particular investment in a specific city is available), and the more SDG indicators may be undefined or outside the boundary of a city or organization (e.g., because a small community does not entertain SDG9-relevant manufacturing or R&D activities).

12. Define the temporal scope of the study.
  a. Identify short-term impacts of technology or infrastructure investments.
    ***Note:*** Short-term impacts include, for example, environmental and socio-economic impacts during a technology’s deployment phase (e.g., air pollution from construction machinery, employment impacts of construction). Smaller-scale investments in energy technologies (e.g., one wind park rather than a complete supply chain) may often be labeled short-term because they are limited to project lead times. Short-term impacts will be particularly relevant when all technology components are imported, and no long-lived manufacturing infrastructure is built locally.
  b. Identify long-term impacts of technology or infrastructure investments.
    ***Note:*** Long-term impacts include system-level environmental impacts and firm-level as well as macroeconomic impacts of technology investments. Long-term impacts will be particularly relevant when components are manufactured locally, as manufactured technologies will require investments in long-lived machines and factories. If life-cycle impact categories are examined, consequential LCA may be more informative about long-term impacts, as it captures future changes in environmental flows (rather than average impacts in the past covered in attributional LCA).

### Scenario definition

**Timing: A few days**

In this step, assumptions are made on the “storyline” of the technology investment. This includes assumptions on the supply chain structure (local manufacturing vs. component import), the investment scale (one project or a suite of projects), and the ownership structure, financing vehicles, and business models chosen.

13. If no well-defined scenario motivates the study, use two extreme scenarios to bound the range of impacts.
  a. Define a component import scenario where all components and materials are imported to the location in which the technology is used.
  b. Define a local manufacturing scenario where all components and materials are produced in the location where impacts are studied.
14. If the protocol is used to assess an investment proposal, extract details from the proposal on where technologies are sourced (supplier names, locations), or use averages representative of local technology markets, e.g., by using impacts from different locations weighted by import shares
15. If using the protocol in ESG assessments, use average company conditions or define scenarios for manufacturing and deployment locations that span the impacts within a company’s supply chain.

**CRITICAL:** Clearly define the chosen scenario (technology or infrastructure deployment location, component supply chain structure, etc.) in the methods section.

### Data collection

**Timing: A few weeks to months depending on data availability**

This step collects data that match the decisions made in steps 2–5, i.e., data enabling the analysis of linkages defined for a particular technology impact category (step 5), linkage conceptualization in terms of direction, degree of causality, weight, and evaluation metrics (steps 7–10), geographical and temporal scope (steps 11 and 12) and investment scenario (step steps 13–15). Data sources and weblinks related to steps 16–18 are provided in key resources table below.

16. Check the SDG Global Database to see whether the SDG indicators of interest (secondary SDG indicators) are available for the country and period of interest.

***Note:*** Background information on individual datasets (definitions, measurement methods) is given in the Metadata Repository (https://unstats.un.org/sdgs/metadata/)

17. Expand your pool of socio-economic data from the SDG Global Database with data from international organizations, including the World Bank, International Labor Organization, and OECD.
  a. For employment by industry and activity, consider the OECD employment database.
  b. For R&D intensities of economies and R&D personnel by industry, consider the OECD’s Research and Development Statistics
  c. For occupational safety statistics, refer to a summary by the International Labor Organization (ILO) of international datasets as well as to country-level sources (e.g., the Bureau of Labor Statistics in the US; the UK Health and Safety Executive; the China Labor Bulletin).
  d. For leadership positions held by women and men, use ILO data and reporting.
18. Expand your pool of environmental data with data from LCA inventories or LCA models, including EcoInvent and GREET (Argonne National Lab).
  a. For EU waste statistics, use data on waste streams provided under the Waste Statistics Regulation.
  b. For air quality data, use data from the World Health Organization or GAIA air quality monitoring stations.

**Table:** 

Data type	Organizations	Website
SDG indicator data	UN, SDG Global Database	https://unstats.un.org/sdgs/dataportal
Employment by industry	OECD	https://data.oecd.org/emp/employment-by-activity.htm
R&D expenditure and personnel by sector and enterprise type	OECD Chinese Statistical Yearbook (Tables 20-x)	https://www.oecd.org/sti/inno/researchanddevelopmentstatisticsrds.htm http://www.stats.gov.cn/sj/ndsj/2020/indexeh.htm
Occupational accidents by industry	International Labor Organization	https://www.ilo.org/moscow/areas-of-work/occupational-safety-and-health/WCMS_249278/lang—en/index.htm
Leadership positions by gender	International Labor Organization	https://www.ilo.org/global/about-the-ilo/how-the-ilo-works/multilateral-system/g20/reports/WCMS_762098/lang—en/index.htm
Life-cycle emissions	GREET, Argonne National Laboratory	https://greet.es.anl.gov/net
	Ecoinvent Database	https://ecoinvent.org/the-ecoinvent-database/
Waste statistics	European Union	https://ec.europa.eu/eurostat/web/waste/data/database
	United States Geological Survey	https://www.usgs.gov/centers/national-minerals-information-center/recycling-statistics-and-information
	Chinese Statistical Yearbook (Tables 26-x) Waste Statistics and related World Bank Report	http://www.stats.gov.cn/sj/ndsj/2020/indexeh.htm https://openknowledge.worldbank.org/server/api/core/bitstreams/0f2590e4-0fdb-5f6f-997a-0d736e6cfaaf/content
Air pollution data	World Health Organization	https://www.who.int/data/gho/data/themes/air-pollution
	GAIA Real Time Air Quality Index	https://aqicn.org/map/world/
	United States Environmental Protection Agency	https://www.epa.gov/air-emissions-inventories/2017-national-emissions-inventory-nei-data
National average water use intensity by country	United Nations	https://sdg6data.org/en/indicator/6.4.1

### Design of impact analysis

**Timing: A few days**

This step involves choosing a conceptual or quantitative method for relating technology investments to SDG indicator changes. Since most SDG indicators are defined at the national level, there is an inherent challenge in assessing the effect of one additional unit of technology-related activity (investments in manufacturing capacity and/or deployment activities) on SDG indicators. However, this challenge can be overcome by taking a ceteris paribus approach (see step 19 below) or making assumptions about system-level impacts and substitution effects of adding an additional unit of technology activity.

19. If you choose the most straightforward ceteris paribus approach employed in,^1^ evaluate the effect of one unit of technology-related activity on an SDG indicator relative to the average performance of the SDG indicator at a particular point in time.

***Note:*** For example, for SDG 9 and indicator 9.5.1 (R&D expenditure as a proportion of GDP), the R&D intensity of industries in a technology’s production network can be compared to the R&D intensity of the country in which the investment in an industry is assumed to be made. All else equal, investing in an industry with a higher R&D intensity should increase the R&D intensity of an economy, while investing in an industry with lower R&D intensity should decrease an economy’s R&D intensity. Where possible, however, comparisons should be made not between initiatives and entire economies but at more granular levels (e.g., changes in the R&D intensity of a specific industry, assuming a scale-up of sectors required for the technology or infrastructure studied).

As another example, for SDG 12 and indicator 12.5.1 (National recycling rate, tons of material recycled out of total waste generated), the recycling rate in the industries within a technology’s production network is compared to a country’s or region’s municipal solid waste recycling rate, as municipal solid waste is the largest waste category by mass. All else equal, investing in technologies with higher recycling rates can thus increase the average recycling rate at the national level.

20. As an alternative approach, consider the type of technology-related activity displaced by an investment to produce more realistic assessments (“ceteris paribus plus substitution”). For example, investments in the upscaling of solar photovoltaics manufacturing may be made instead of investments in industries that supply components for more carbon-intensive energy technologies, and solar electricity may displace more carbon-intensive electricity sources.

***Note:*** Considering substitution will require additional data collection and modeling to understand how technology investments are incentivized (e.g., a shift in government support for market expansion policies away from one and towards another technology may suggest substitution) or how supply curves of new technologies compare to existing ones (e.g., the high availability of solar PV during peak demand periods makes the substitution of fossil-fired peaking plants more likely). In general, considering the substitution of existing with new technologies will likely make SDG linkages appear stronger, because rather than just adding one unit with improved performance to the population, another unit with worse performance is retired. Thus, considering the impacts of technology substitution can be crucial in studies of weighted linkages where ceteris paribus weights for different linkages are similar.

### Application of impact analysis

**Timing: A few weeks to months depending on the assessment method.**

This step assesses the potential of technology industries to influence SDG indicators. It computes a set of metrics describing how many of the potential linkages between technology industries and SDG indicators show potential for positive or negative impacts of investments supporting the primary SDG on the secondary SDGs (co-benefits and trade-offs).

21. Assess the potential of technology industries to influence SDG indicators using literature review (as done in^1^) or expert assessment.
  a. In both cases, define criteria for setting a potential link to “1” (potential for co-beneficial link), “‒1” (potential for trade-off link), or 0 (negligible potential for direct influence of a technology industry on an SDG indicator), e.g., the number of existing studies supporting a potential relationship between technology investment and SDG indicator changes, or the level of expert agreement (several experts in a group of experts, or one expert strongly agreeing).
    i. For studies using literature review, use the industry name and the SDG indicator name for keyword searches.
    ii. Define a criterion (e.g., one or several peer-reviewed studies documenting a mechanism through which an industry or service affects an indicator).
  b. Go through the matrix of technology impact categories (e.g., industries) and SDG indicators and fill each row with ‘‒1’, ‘1’, or ‘0’. For a template, refer to Table S1, ‘Technology SDG network’, tabs ‘Matrix for Figure 4’ and ‘Matrix for Figure 5’ for the matrices used in Klemun 2023^1^ (see key resources table, Deposited Data, for the download information).
22. For each combination of technology and SDG indicator, compute a technology-indicator density *d*_*i*_ to measure the number of connections with documented potential for influence (i.e., those set to 1 or ‒1 in step 21b) over the total number of possible connections (*n*):
  a. Compute technology indicator densities separately for potential co-benefits and trade-offs to separate positive and negative impacts of technology investments (and avoid masking negative impacts with positive ones).
23. For each combination of technology and SDG, define an aggregated density metric (linkage density *d*) that gives the number of total industry-indicator linkages showing potential for positive or negative impacts over the total number of possible industry-indicator linkages:
  $$d=\frac{1}{s}\;\sum_{i=1}^{s}d_{i}\text{.}$$
  Compute *d* separately for co-benefit (co-benefit linkage density) and trade-off linkages (tradeoff linkage density).
  a. The number of total industry-indicator linkages showing potential for positive or negative impacts results from step 22.
  b. The total number of possible industry-indicator linkages is equal to the number of industries in a technology’s supply network times the number of indicators assigned to an SDG.
  c. Refer to Excel file ‘Technology SDG network’, tabs ‘Figure 4 Data’ and ‘Figure 5 Data’ for the linkage densities shown in Figures 4 and 5 in Klemun 2023.^1^
24. Visualize the results by plotting linkage densities on the y-axis separately for each SDGs and technology (x-axis). An example is shown in Figures 4 and 5 in Klemun 2023.^1^
  a. Co-benefit densities can be shown as positive values between 0 and 1.
  b. Tradeoff densities can be shown as negative values between 0 and ‒1.

***Note:*** You can interpret unweighted linkage densities as a partial measure for network connectivity. The densities measure what fraction of possible links may actually make a contribution to relationships between two SDGs (a full measure would also incorporate linkage weights).

### Robustness tests

**Timing: A few days to weeks**

This step tests the robustness of your conclusions to uncertainties in the input data and assumptions. A few options are outlined below. Robustness tests are particularly important when results are non-conclusive, i.e., linkage densities across SDGs are similar.

25. Random weight assignments: Using unweighted linkages is equivalent to drawing link weights from discrete, uniform distributions. One approach to test the robustness of your conclusions is to replace unweighted linkages with link weights drawn randomly from a continuous distribution, for example. This can be done repeatedly to check if overall rankings of linkage densities are affected by link weights.
26. Repeat the analysis for weak results with data from other countries to check whether a conclusion is context-specific or holds more generally.
27. When using expert elicitation or stakeholder groups to rate linkages and derive weights, consider repeating the analysis in different groups (i.e., having at least two groups come up with weights for the same links), allowing participants to re-examine and potentially revise their judgments (to reduce the impact of contextual factors), and comparing expert/stakeholder weights to weights randomly drawn from a distribution.

### Deriving recommendations for SDG linkage enhancement

**Timing: A few days**

This step uses the results from “application of impact analysis” to devise strategies for linkage enhancement.

28. Linkage densities of zero can indicate that a) effects of technology investments on SDG indicators are implausible or negligible, b) that they are not documented in the literature or recognized by experts yet, or c) that the strict definition of linkages as functional led to an omission of impacts that depend on how a technology is deployed and used, but are not strictly required for its use or a direct consequence thereof (e.g., scaling low-carbon technology could affect SDG2 due to competition for farmland).
  a. Revisit the reasons and modeling choices that led to linkage densities of zero, to distinguish highly implausible from context-dependent linkages
    i. For context-dependent linkages, make recommendations that make these use cases more likely (e.g., subsidies for the use of renewable electricity in agriculture or emissions standards for agricultural machines).
29. Linkage densities between 0 and 1 indicate that there is potential for co-benefits between two SDGs. However, this potential may still need to be fully developed as it does not accrue across all technology impact categories (e.g., technology industries) and/or does not affect all SDG indicators. (If all impact categories were to affect all indicators, the density would equal 1.)
  a. If not all impact categories contribute, and if the impact categories are industries, search for SDG-relevant innovations in these industries (mining, materials processing, manufacturing, construction etc.) broadly and consider whether these could be applied in the industries as used by a particular technology (e.g., wind turbine materials processing, wind turbine construction).
30. Linkage densities between 0 and ‒1 indicate that there is potential for trade-offs between two SDGs.
  a. Follow the steps outlined under Step 29 but consider measures and innovations that mitigate technology-related impacts rather than strengthening them.

***Optional:*** The protocol may produce a large number of recommendations, raising questions about prioritization. Consider grouping the final list of recommendations into low-, medium- and high-priority recommendations based on multi-criteria decision analysis, expert input, or the interconnectedness of SDGs amongst all SDG targets (where high-priority recommendations are labelled as such because the SDGs they relate to are central to the achievement of other SDGs).

## Expected outcomes

The anticipated outcomes of the protocol include: an assessment of potential linkages between technology impact categories and SDG indicators for each combination of technology (used to support a primary SDG) and SDG in the set of secondary SDGs of interest; a set of indicator-specific and overall SDG-level metrics for co-benefit and trade-off linkage densities. For example, if there is one primary SDG and all other SDGs are secondary SDGs, the protocol will produce 16 positive and 16 negative aggregate scores for each technology (see Figures 4 and 5 in Klemun 2023^1^). In terms of further interpretation, if a technology exhibits higher linkage densities than another one across multiple SDGs, that result may indicate that this technology is particularly likely to have simultaneous effects on other, non-primary SDGs (and thus well suited for early economic development stages, when indicators for all SDGs tend to be weak).

For weighted linkages, a set of results on linkage strength and qualitative conclusions on weaker, stronger, and uncertain linkages.

Further outcomes include: a list of industries and services (or other impact categories) contributing to the scores (linkage densities) mentioned above (see Figures 3 and 6 in Klemun 2023^1^); a visualization of co-benefit and tradeoff densities across all technologies and SDGs considered; a visualization of SDG impacts at the level of individual impact categories; a set of final, high-level results on positive and negative linkages between SDGs; a set of recommendations for enhancing co-benefit linkages and mitigating trade-off linkages.

## Quantification and statistical analysis

This protocol is mainly focused on unweighted SDG linkages. However, future work and specific policy and investment contexts may call for linkage quantifications to distinguish stronger from weaker linkages and enable prioritization. If quantification is of interest, you can explore the following research directions.

• **Direct modeling:** If data are available for model calibration and change mechanisms are known, direct modeling of indicator changes in response to technology investments is one approach (e.g., emissions reductions from the displacement of high- with low-carbon electricity);
  ○ For example, to estimate the strength of relationships between SDG7 and SDG11 (“Sustainable Cities and Communities”) due to the expected effects of clean energy technology investments on indicator 11.6.1 (“Annual mean levels of fine PM in cities”), you can proceed as follows:
    - Use location-specific emissions inventories to derive average fine particulate matter (PM 2.5) emissions factors for local electricity generation sources.
    - Compute adjusted PM 2.5 emissions factors for power generation in that location, assuming that a fraction of electricity generation will be displaced by zero-PM solar electricity generation. Use location-specific emission factors where possible.
    - Compute the expected change in total PM 2.5 levels given a range of projections from the literature for the future contributions of road transport, air traffic, marine navigation, and other combustion sources. Emissions can be linked with ambient concentrations using reduced-complexity air quality models, for example.
  ○ Or, to estimate the strength of the linkage between clean energy technology investments to pursue SDG7, and SDG3 indicator 3.9.1 (“Mortality rate attributed to ambient air pollution”), you can proceed as follows:
    - Collect data from the literature on the effects of historical changes in air pollution on mortality in the location of interest (e.g., percent changes in mortality due to a given unit increase in air pollution indices) or a location with comparable sociodemographic characteristics.
    - Use the resulting relationship to estimate the reduction in specific mortality types (e.g., cardio-respiratory) expected due to the projected air pollution reduction.
    - If possible, use projections for the future population structure of the study region as a basis and adjust calculations from the literature for population aging and changes in vulnerability to air pollution-related disease and death.
  ○ Or, to estimate changes in indicator 15.1.1 (“Forest area as a percent of total land area”) due to energy infrastructure installations, you can proceed as follows:
    - First, collect data on forest coverage (e.g., World Bank, satellite data^18^).
    - Either draw on technology-specific land use estimates from the literature or use engineering relationships between the output power of the power plant, plant efficiency, and the associated necessary size of the plant to estimate the expected forest area reduction if forest sites are chosen (as the worst-case scenario).
• **Expert elicitation:** Inviting experts to rate the unweighted linkages derived from the literature is another possible approach.
  ○ To allow informed assessments using all available information from the initial research, experts should be given access to matrices containing industry and service impacts on individual SDG indicators or qualitative summaries of how different industries and indicators contribute to linkage density results. That way, experts can directly assess the mechanisms likely to shape individual linkages rather than rating “black box” linkage density results.
  ○ Experts can also be consulted in conjunction with direct modeling efforts (e.g., to assess the plausibility of modeling results from the perspective of a particular domain, such as air pollution modeling, health, and demography, or industrial development).
• **Focus groups:** If local perspectives are of particular interest, consulting stakeholders in focus groups is another approach.
  ○ Use focus groups when hypotheses exist on potential SDG linkages not covered by the industry-indicator relationships derived in steps 21–24. For instance, communities may expect compensation for carrying the environmental burden of new energy infrastructure or may have esthetic or noise concerns. Focus groups may help identify technology impact categories (industry, service, other categories) most closely associated with these concerns, pointing to potential indicators that should be added to a standard SDG indicator set.
  ○ Use focus groups to identify linkages important to local communities, either because they are more directly affected by those linkages or care about them for cultural and political reasons.

## Limitations

### Expert consultation and other methods for linkage weight elicitation

While this protocol mentions expert elicitation and focus groups as potential methods for deriving linkage weights, it does not provide step-by-step guidance for applying these methods. Interested protocol users can refer to the literature for further information on specific practices like Delphi processes^19^ and modified versions thereof^20^ or focus group methods.^21^ In addition, a review of methods applied in academic studies of SDG linkages can be found in Bennich 2020.^22^

### Expansion of SDG indicators

This protocol provides a few examples of how one might identify additional SDG indicators not included in the current UN indicator framework, e.g., by starting from LCA impact categories and identifying categories unmatched by SDG indicators. However, as discussed in Klemun 2023,^1^ a broader consideration of sustainability-related technology characteristics (scalability, equity-related characteristics) could be used to identify a more extensive set of relevant, technology-centric metrics in future research. In other words, technology and infrastructure investments may affect SDGs in additional ways currently not captured by indicators, which is a promising area for future research and protocol development.

### Data gaps

While this protocol points to data sources associated with individual SDGs, it is not based on a comprehensive SDG indicator data availability assessment. The protocol, therefore, does not distinguish between easy-to-access data and potential data gaps that may be relevant to protocol users working in specific technology domains or world regions. Future work could include a broader set of technology- and region-specific use cases to assess data availability better and provide more detailed guidance to protocol users.

### Model validation

This protocol does not describe steps to validate protocol outputs. One reason is the lack of similar studies (i.e., studies that also evaluate impacts of technologies on SDG indicators and SDG linkages) and the resulting limits to comparative validation. Empirical validation may be possible in future work by collecting indicator data from locations with large-scale historical adoption or dominance of certain technologies.

## Troubleshooting

### Problem 1

If data gaps are identified in Steps 16–18 (‘data collection’) (e.g., no data are available for industry impacts and indicators of secondary SDGs of interest), consider using proxy data from other industries and locations.

### Potential solution

• Advance in your search for proxy data step-by-step from the next best data option to the worst option.
  ○ For example, when decomposing technologies into industry and service impacts, data may not be available specific to that industry or service in a location (e.g., there is no employment or recycling data for metal industries in individual countries).
    - As a next best option, search for data from a similar industry (e.g., within the same 4- or 2-digit NAICS code category) in the exact location.
    - As another option, search for data from the specialized industry or service from another country with similar socio-economic conditions.
• Proxy locations should be selected from the same world region (e.g., use data from one EU country instead of another) due to regional similarities in socioeconomic development, environmental regulation, and compliance.
  ○ When baseline data on current values of SDG indicators in locations of interest is not available, consider using historical data from the exact location or global as well as regional averages representing a more recent year.

### Problem 2

If selecting technologies or fuels for analysis (Step 2) that are used in larger infrastructure systems or require ancillary infrastructure to function (e.g., software running on a personal computer using cloud data), infrastructure systems impacts need to be attributed to the specific technology or fuel of interest to avoid under- or overestimation of impacts.

### Potential solution

• Include systems impacts and ancillary infrastructure in the analysis, and account for the usage share of the technology analyzed (e.g., fraction of natural gas in systems co-producing natural gas and oil, share of total asset lifetime, throughput, or daily operational time that it is used for the technology analyzed).
  ○ For example, a direct air capture plant will require CO_2_ transport and storage facilities (or an industrial user) to achieve negative emissions. However, neither trucks, pipelines nor storage facilities are typically built for just for one single plant. Thus, when quantifying linkage strengths, multiply the total system impact by the share of plant-specific CO_2_ in total CO_2_ throughput during a particular time period to generate an estimate for the additional, infrastructure-related impacts.
  ○ An example for attributing emissions from oil and gas co-production is given in Klemun 2019,^23^ Supplementary Material section 1.

## Resource availability

### Lead contact

Further information and requests for resources and reagents should be directed to and will be fulfilled by the lead contact, Magdalena Klemun (magdalena@ust.hk).

### Materials availability

This study has generated no new materials.

### Data and code availability

Sources of raw data used in this study have been listed in the key resources table or references in Klemun 2023^1^ (main paper or SI). The data and main analytical steps to generate the study’s key results (Figures 4 and 5) are available in Excel (see Table S1 listed in key resources table).

## Supplemental information

Table S1. Technology SDG network, related to step 21
